# Crimean–Congo Hemorrhagic Fever Virus Infections in Slaughtered Camels and Abattoir Workers in the United Arab Emirates

**DOI:** 10.1155/tbed/3409106

**Published:** 2025-05-08

**Authors:** Mohamud Sheek-Hussein, Aboma Zewude, Aminu S. Abdullahi, Jamila Al Neyadi, Babiker Osman, Amir Abdullah Hassen, Hassan Zackaria Ali Ishag, Abraham Nii Okai Commey, Mohamed Saleh A. L. Breiki, Asma Abdi Mohamed Shah, Mervat Mari Al Nuaimat, Kaltham Kayaf, Mohamed Elfatih Hamad, Ahmed R. Alsuwaidi, Robert Barigye, Balázs Ádám, Gobena Ameni

**Affiliations:** ^1^Institute of Public Health, College of Medicine and Health Sciences, United Arab Emirates University, Al Ain City, Abu Dhabi Emirate, UAE; ^2^School of Public Health, Loma Linda University, Loma Linda, California, USA; ^3^Department of Veterinary Medicine, College of Agriculture and Veterinary Medicine, United Arab Emirates University, Al Ain City, Abu Dhabi Emirate, UAE; ^4^Al Bawadi Abattoir, Al Ain Municipality, Al Ain City, Abu Dhabi Emirate, UAE; ^5^Biosecurity Affairs Division, Development and Innovation Sector, Abu Dhabi Agriculture and Food Safety Authority, Abu Dhabi City, Abu Dhabi Emirate, UAE; ^6^Animal Development and Health Department, Ministry of Climate Change and Environment, Dubai City, Dubai Emirate, UAE; ^7^Department of Pediatrics, College of Medicine and Health Sciences, United Arab Emirates University, Al Ain City, Abu Dhabi Emirate, UAE

**Keywords:** abattoir workers, Crimean–Congo hemorrhagic fever, dromedary camels, molecular detection, seroprevalence, United Arab Emirates

## Abstract

Crimean–Congo hemorrhagic fever (CCHF) is a tick-borne disease caused by the CCHF virus (CCHFV) and is characterized by the sudden onset of high fever and hemorrhagic manifestations. This study aimed to estimate the seroprevalence of anti-CCHFV antibodies in dromedary camels and workers at the Al Bawadi abattoir in the United Arab Emirates (UAE). In addition, the camels and human subjects were screened for CCHFV RNA, and the knowledge level of abattoir workers regarding CCHF zoonosis was assessed. A cross-sectional study was conducted between March 2022 and June 2023 at the Al-Bawadi abattoir in Al Ain with 393 camels and 86 abattoir workers. Anti-CCHFV immunoglobulin G (IgG) antibody was tested in camel sera using a multispecies indirect enzyme-linked immunosorbent assay (ELISA). The sera of abattoir workers were tested for anti-CCHFV IgG antibody using a human CCHFV ELISA kit. Camel and human serum samples were tested by reverse transcription quantitative real-time polymerase chain reaction (RT-qPCR) to detect CCHFV RNA. A questionnaire survey was used to evaluate abattoir workers' knowledge of the risk of zoonosis of CCHF. The seroprevalence of anti-CCHFV antibodies in camels slaughtered at the Al-Bawadi Abattoir was 65.1% (95% confidence interval [CI]: 60.4%–70%). However, the RNA prevalence was only 1% (4/393), with cycle threshold (Ct) values ranging from 34.58 to 38.21. The seroprevalence of anti-CCHFV antibodies in abattoir workers was 29.1% (95% CI: 20.3%–40.4%), but none of the abattoir workers tested positive by RT-qPCR. Seropositive abattoir workers had a longer duration of working in the abattoir (median = 10 years; interquartile range [IQR]: 6.0–14.0) than seronegative abattoir workers (median = 7.5 years; IQR: 5.0–14.0) although the difference was not significant (*p* > 0.05). Most abattoir workers (73%) knew that CCHF is zoonotic. The seroprevalence of anti-CCHFV antibodies was high in both camels and abattoir workers at the Al-Bawadi Abattoir, and viral RNA was detected in four camels. Hence, active surveillance and reinforcement of control measures are recommended.

## 1. Introduction

Crimean–Congo hemorrhagic fever (CCHF) is a zoonotic tick-borne disease caused by the CCHF virus (CCHFV). In humans, the illness is characterized by sudden onset of high-grade fever, chills, severe headache, dizziness, and back and abdominal pain. In addition, severe CCHF may demonstrate hemorrhagic manifestations ranging from petechiae to large areas of ecchymosis. The fatality rate of CCHF in humans has been estimated to be 30% or higher [1]. In contrast to humans, CCHFV infections in animals do not cause clinical disease, and thus, it does not affect livestock animal production [2]. However, domestic and wild animals serve as asymptomatic hosts and amplify the virus and thus serve as sources of infection for human occupational groups including animal handlers, slaughterhouse workers, and agricultural workers at high risk of exposure in endemic areas.

Humans are infected through the bite of an infected tick or exposure to blood or tissue from infected animals; human-to-human transmission, particularly in healthcare settings, has been reported [3]. The number of human cases of CCHF is estimated to be 10,000–15,000 worldwide each year, although it is difficult to know the definitive number of cases since the majority of the cases are thought to be subclinical or occur in locations with limited disease surveillance or laboratory testing capability [4]. The natural vectors of CCHFV are *Hyalomma* ticks, and as a result, the global distribution of the virus coincides with the distribution of *Hyalomma* ticks [5, 6]. The natural transmission cycle of CCHV includes transovarial (vertical) and transstadial (horizontal) in among ticks and a tick-vertebrate-tick cycle involving a variety of wild and domestic animals [2]. Humans are considered dead-end or accidental hosts for the virus as they are not the source of infection for ticks [4]. *Hyalomma* ticks feed on a variety of domestic ruminants and wild herbivores. However, although infection of these animals with CCHFV is subclinical, the associated viremia levels are sufficient to enable the virus transmission to uninfected ticks [7]. In addition to *Hyalomma* ticks, which are the main vectors' of CCHV, *Rhipicephalus* and *Dermacentor* genera have also been reported to be vectors and reservoirs of CCHFV [8, 9].

CCHFV was first recognized during the mid-1940s during World War II when 200 Soviet Union soldiers stationed in the Crimean Peninsula developed hemorrhagic fever after exposure to ticks [10], which lead to the naming of the etiologic virus as Crimean hemorrhagic fever virus. Later, in 1956, an outbreak of a similar hemorrhagic fever occurred in humans in the current Democratic Republic of Congo [11], as a result of which the causative virus was named the Congo hemorrhagic fever virus. In the early 1970s, the names of the two viruses were merged and named CCHFV after the two viruses were found to be serologically indistinguishable [4]. Since then, several studies have demonstrated that the disease is widely distributed throughout Africa, the Middle East, Southeast Asia, and Southern and Eastern Europe, closely following the wide geographical distribution of its host, the *Hyalomma* tick [12]. Furthermore, studies reported a long-range transportation spread of the virus by birds infested with infected ticks, trading of livestock infested with infected ticks, and expansion of the tick to new areas because of climate change [13–15]. Expansion of CCHFV to new geographic regions poses a risk to the new naïve human population and is considered a public health threat, as there is no licensed vaccine or specific antivirals exist to prevent or treat CCHF so far [1].

CCHFV is an enveloped, multisegmented, single-stranded negative-sense RNA virus belonging to the *Orthonairovirus* genus, order Bunyavirales and family Nairoviridae [16]. The virus genome has three single-stranded segments that are characterized by a complex replication cycle, which is prone to error and reassortment, thereby leading to the generation of genetically diverse strains and the emergence of novel variants [4]. The three segments of the RNA genome of CCHFV include the small segment (S) segment, which encodes the nucleocapsid protein and nonstructural protein; the medium (M) segment that encodes membrane glycoproteins and several nonstructural proteins, and the large (L) segment, which encodes RNA-dependent RNA polymerase [17, 18].

Although CCHF is clinically inapparent in domestic and wild animals, screening these animals for anti-CCHFV antibodies can serve as a good indicator of the circulation of the virus in specific region or country [2]. Such investigations further allow the identification of high-risk areas for human infection. In the United Arab Emirates (UAE), studies conducted over the past 40 years have shown that CCHF is endemic in the UAE [19–23]. Although these studies have underscored the potential risk that the disease poses to human health, additional multidisciplinary studies involving humans and livestock are required to complement previous studies and to establish the epidemiology and zoonotic significance of CCHF in the country. Similar to the greater Middle Eastern region, camels are considered an important livestock species in the UAE, where they serve as a source of meat and milk. Regarding the zoonosis of CCHF, abattoir workers are among the demographic segments of the population that are at the highest risk of infection with CCHFV because of their constant exposure to the blood and body fluids of potentially infected livestock species [24–28]. Therefore, the objective of this study was to estimate the seroprevalence of anti-CCHFV antibodies and detect CCHFV RNA in camels and abattoir workers at the Al-Bawadi abattoir in Al Ain City, UAE, and to assess the knowledge of the abattoir workers regarding the zoonotic nature of CCHF.

## 2. Materials and Methods

### 2.1. Study Setting

**Figure 1:**
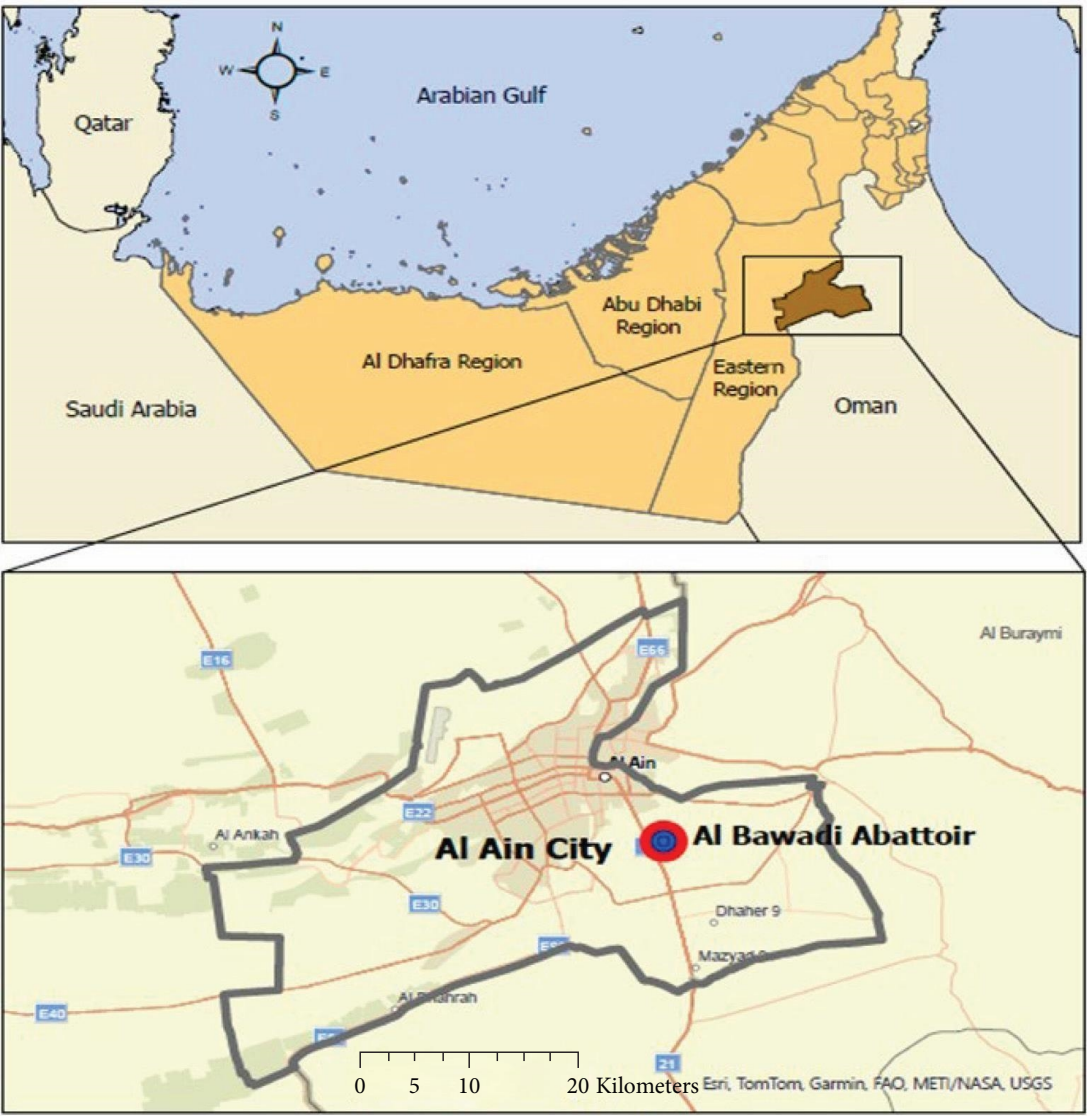
Map of the study area. The top map shows the location of Al Ain city in the United Arab Emirates, while the bottom map shows the location of the Al Bawadi abattoir in Al Ain city.

The study was conducted at the Al-Bawadi abattoir, which is located on the outskirts of Al Ain city in the Emirate of Abu Dhabi, UAE (Figure 1). The majority of the camels included in the study were brought to the slaughterhouse from different locations in the UAE. Some camels studied were imported from Oman. However, owing to the lack of consistent record-keeping, the research team could not definitively determine the exact origin of each study camel. However, earlier records showed that 98.2% of the 28,442 camels slaughtered at the Al-Bawadi abattoir were originally from the UAE, suggesting that nearly all the camels used in this study originated from the UAE. The livestock market is located next to the Al-Bawadi abattoir, which serves as a source of camels for the abattoir.

### 2.2. Study Design, Sample Size, and Sampling

This cross-sectional study was conducted between October 2022 and August 2023. Assuming that all the study camels lived in the UAE before they were recruited, the sample size was estimated by considering the total camel population of the UAE as 450,000, the expected prevalence of CCHE as 50%, and a 5% margin of error with a 95% confidence interval (CI). Accordingly, 384 camels were required for the study, and 393 camels were recruited. Systematic sampling was used to select camels from those that were slaughtered daily. For the human study, of the 110 workers in the Al Bawadi abattoir, 86 expressed their willingness to participate in the study. The study subjects were also used for the descriptive study published on the *Coxiella burnetii* infection in camels and abattoir workers by the same team of researchers [29].

### 2.3. Collection of Animal Data and Blood Samples

During the ante-mortem examination of the camels, data on age, sex, and body condition were collected. Body condition was scored based on previously published protocols [30–32]. Fat levels at different anatomical sites were evaluated to estimate the body condition of the camels. The fat coverage of each camel was assessed based on (1) the visibility of the ribs, the ischiatic and coxal tuberosities, and the scapula; (2) the extent of the hollowness of the flank; (3) the extent of the deepness of the recto-genital zone; and (4) the circumference and height of the hump. Based on these criteria, the body scores of the camels were broadly grouped into three categories based on body condition: thin, medium, and fat.

The ages of the camels were estimated based on the status of their teeth because there were no records of their age. The ages of the camels were estimated according to a previously published protocol [33]. The protocol considers the eruption and wear of camels' teeth. According to these authors, all the deciduate teeth erupt at 9 months of age, while at 4 years, all the deciduate incisors and canines wear down. Furthermore, all the permanent incisors and canine teeth erupt at 7 years, and at 12 years, they start wearing and wear down at about 15 years. At 20 years of age, all the permanent teeth are down and clearly separated from each other.

Blood samples were collected from the jugular veins of the camels in 5 mL plain vacutainers for serum extraction prior to slaughter. Additionally, 86 abattoir workers from the same slaughterhouse were recruited for the study, and 5-mL blood samples were collected from the cephalic vein for serum extraction.

### 2.4. Questionnaire

A one-page questionnaire was prepared in English and translated into Arabic. The abattoir workers verbally consented to be questioned by the researchers. The questionnaire collected information on their demographics, animal contact, occupational injuries, workplace preventive measures (use of personal protective equipment [PPE]), and knowledge of zoonotic diseases and their routes of transmission between animals and humans.

### 2.5. Double Antigen Enzyme-Linked Immunosorbent Assay (ELISA) for Detection of Anti-CCHFV Antibodies in Camel Sera

Serum was extracted from camel blood samples by centrifugation and then tested for CCHFV reactive antibodies using the ID Screen CCHF Double Antigen Multispecies ELISA kit (ID VET Innovative Diagnostic, ID Screen, Montpellier, France) following the manufacturer's instructions. The microtiter plate wells were coated with recombinant purified CCHFV nucleoprotein antigen, and 30 μL of the serum samples along with 30 μL of the positive and negative controls were added. The plates were incubated at room temperature for 45 min, after which they were washed appropriately with the diluted kit wash buffer. After washing, 50 μL horseradish peroxidase (HRP)-conjugated recombinant purified CCHFV nucleoprotein was added to each well and incubated for 30 min at room temperature. After washing off the excessive conjugate, 100 μL substrate solution (TMB) was added to each well, followed by 100 µL of the termination solution after 15 min. The presence of anti-CCHF antibodies was determined by evaluating the optical density (OD) at 450 nm of the microtiter wells using a spectrophotometer (BioTek Instrument Inc., Highland Park, USA). Test results were interpreted according to the manufacturer's instructions. After the OD readings were obtained, the validity of the readout was checked for each plate. Based on the manufacturer's recommendations, the test is considered valid if the mean OD value of the positive control (OD_PC_) is >0.350 and the ratio of the mean OD value of the positive and negative controls (OD_PC_/OD_NC_) is >3.0. Following confirmation of the validity of the results for each plate, the results were interpreted individually. Briefly, the sample percentage (S/P%) was calculated as follows: S/P% = (OD_450_ sample)/(mean OD_PC_ 450) × 100. Any sample with S/P% ≤ 30% was considered negative, while any sample with S/P% >30% was considered positive. Positive samples were further grouped into low (S/P% = 30%–99.9%), medium (S/P% = 100%–130%), and high (S/P% > 130%), depending on the intensity of the OD_450_ values.

### 2.6. Human CCHF ELISA

A sandwich ELISA was used to detect CCHFV particles in the sera of abattoir workers. CCHFV reactive antibody precoated microplates were used for running the assay. Briefly, the setting of the wells (two positive controls, two negative controls, a test sample, and a blank well) was performed according to the manufacturer's instructions (Abbexa Ltd, Cambridge, UK; website: www.abbexa.com). Then, 50 μL of negative control and 50 μL of positive control were added into their designated wells, while 50 μL of sample diluent was added into the zero (blank) well. Similarly, 50 μL of human serum samples diluted 1:5 in the kit wash buffer diluent were added into the test wells, and the plates were covered and incubated at 37°C for 30 min. Following this step, the plates were washed five times with 1× wash buffer. Thereafter, 50 μL of HRP-conjugated secondary antibody was added to each well, except for the blank wells, and incubated at 37°C for 30 min. The plates were removed from the incubators and washed five times with 1× wash buffer. Thereafter, 50 μL of TMB substrate A and 50 μL of TMB substrate B were added to each well, mixed, and incubated at 37°C for 15 min. This step was followed by the addition of 50 μL of stopping solution into each well, and the OD_450_ readings were immediately measured using a spectrophotometer (BioTek Instrument Inc., Highland Park, USA). The validity of the tests was checked based on the recommendation of the kit manufacturer, whereby the test was considered valid if the mean OD_450_ value of the positive control was ≥1.0 and the OD_450_ value of the negative control was ≤0.20. The cutoff value was calculated as the mean OD value of the negative control +0.15, and samples with OD_450_ values less than the cutoff were considered negative, while samples with OD_450_ values at the cutoff or higher were considered positive. The positive samples were further grouped into low (OD = 0.30–0.99), medium (OD = 1.0–1.99), and high (OD ≥ 2), depending on the intensity of OD values.

### 2.7. Detection of CCHFV RNA by Reverse Transcription Quantitative Real-Time Polymerase Chain Reaction (RT-qPCR)

RT-qPCR was performed to detect CCHFV particles in serum samples from camels and abattoir workers. To this end, 200 μL of each serum sample was inactivated by heating to 56°C for at least 30 min [34]. Total nucleic acid was extracted from heat-inactivated serum (*n* = 480) using the Taco DNA/RNA Extraction Kit (GeneReach Biotechnology, Taiwan) and Taco Nucleic Acid Automatic Extraction System (GeneReach Biotechnology, Taiwan) as per the manufacturer's instructions. The presence of CCHFV RNA was detected by qPCR using the same primers and probes as previously described [35] with minor modifications. In brief, the PCR master mix (QuantiFast Probe RT-PCR kit [Qiagen]) was prepared in a total volume of 10 μL. The reaction consisted of 7.5 μL of QuantiFast Probe RT-PCR Master Mix (2×), 4 μL of reaction buffer (5×), 0.15 μL of QuantiFast RT Mix, and 0.8 μL of each primer (10 pmol/μL) and 0.3 μL of each probe (10 pmol/μL). Template RNA was added at 5 μL for a total volume of 15 μL. The mixture was placed in a BioRad CFX 96 thermocycler (BioRad) with the following cycling conditions: reverse transcription (50°C for 15 min), enzyme activation (95°C for 15 min), and 40 cycles of amplification (95°C for 15 s and 58°C for 15 s).

### 2.8. Data Management and Analysis

Statistical Package for Social Sciences (SPSS) version 28.0 (IBM Corp 2021, Armonk, NY, USA) and GraphPad Prism 9.4.1 (681) version (GraphPad Software, Boston, MA, USA) were used for data analysis. Qualitative variables were summarized using frequencies and percentages, whereas quantitative variables were summarized using medians and interquartile ranges (IQRs) owing to skewed distributions. Factors associated with CCHFV infection were evaluated using the chi-square and Fisher's exact tests for categorical variables and the Mann–Whitney *U* test for continuous variables. One-way ANOVA was used to compare the mean OD values of the three groups of seropositive camels as well as the three groups of seropositive abattoir workers. Kruskal–Wallis ANOVA and the Mann–Whitney *U* test were used to examine the association between categorical variables and abattoir workers' knowledge scores. Regarding the questionnaire, each correctly answered question was assigned a score of one, adding up to a maximum possible score of 14. Individual scores were then added for each participant to obtain the overall score. Statistical significance was set at <5%.

### 2.9. Ethics Approval and Consent to Participate

Both the human and animal studies were approved by the Abu Dhabi Health Research and Technology Ethics Committee (ADHRTC) with a Ref No. DOH/CVDC/2022/1136 on 4 July 2022. ADHRTC is composed of experts responsible for human ethics and animal ethics. Furthermore, informed consent was obtained from all abattoir workers included in the study. Based on informed consent, 86 abattoir workers participated in the study out of the 110 workers in the abattoir. In addition, all the methods used in animal and human studies were carried out in accordance with the relevant guidelines and regulations. On top of these, an approval was obtained from the Al Ain city Municipality (reference No. AAM/PSH/OUT/2022/2191).

## 3. Results

### 3.1. Seroprevalence of Anti-CCHFV Antibodies in Camels Slaughtered at Al-Bawadi Abattoir

The prevalence of CCHFV antibodies in the camels at the Al-Bawadi Abattoir was 65.1% (95% CI: 60%–70%). No association was observed between the seroprevalence of CCHFV antibodies and sex, age, or body condition of the camels in both univariable and multivariable binary logistic regression analyses (Table 1).

**Figure 2:**
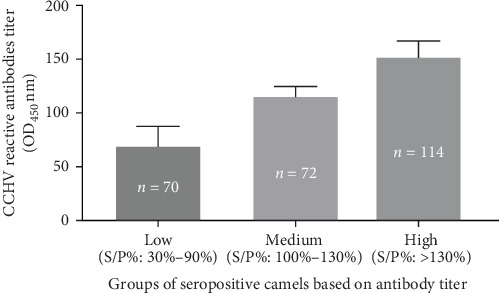
The mean sample percentage (S/P% at OD_450_) of anti-CCHFV antibody titer in slaughtered camels at the Al-Bawadi abattoir. The mean percentage (S/P%) at OD_450_ (mean ± SEM) of positive camels with low, medium, and high antibody titer were 69.54 ± 2.16, 115.8 ± 1.04, and 152.3 ± 1.35, respectively.

The result of ELISA test for anti-CCHFV antibody in slaughtered camels was expressed as the sample percentage (S/P% OD_450_) and presented in Figure 2. Based on the cutoff established by the kit manufacturer, 256 of the 393 camels were seropositive, while the remaining 137 were seronegative. The sample percentage OD_450_ (S/P%) values of the positive camels were grouped as low (S/P% = 30%–99.9%), medium (S/P% = 100%–130%), and high (S/P% > 130%) based on the intensity antibody titer. The mean S/P% (mean ± SEM) of seropositive camels with low, medium, and high antibody titer were 69.54 ± 2.16, 115.8 ± 1.04, and 152.3 ± 1.35, respectively.

The proportions of the seropositive camels with low, medium, and high anti-CCHFV antibody titer were 27% (70/256), 28% (72/256), and 45% (114/256), respectively. Accordingly, the majority (45%) of the seropositive camels had high antibody titer.

### 3.2. Seroprevalence of Anti-CCHFV in the Abattoir Workers

A total of 86 abattoir workers participated in the study, with a median age of 36 years (IQR = 30–41 years), and the majority (83%) were butchers. The median number of years of experience was eight (IQR = 6, 14). Among the sampled workers, Pakistanis (42%) and Bangladeshis (24%) constituted the highest and second-highest nationalities, respectively. Approximately 5% and 7% reported being affected by a zoonotic disease and knowing co-workers affected by a zoonotic disease, respectively (Table 2). All the participants (100%) reported using PPE, whereas none reported a history of vaccination since their employment as abattoir workers.

The overall prevalence of CCHFV antibodies among the human study participants was 29.1% (95% CI: 20.3–40.4). Although not statistically significant, Egyptians (55%) had the highest prevalence compared with other nationalities. Moreover, the seropositive abattoir workers had more years of experience than the seronegative workers (10 years versus 7.5 years) (Table 3). The overall median OD_450_ value of the screened workers was 0.22 (IQR = 0.19, 0.36), with Egyptian workers having significantly higher (*p*  < 0.05) antibody titer than the other nationalities.

**Figure 3:**
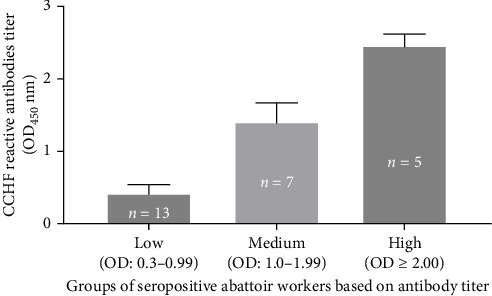
Mean optical density (OD) of anti-CCHFV antibody in abattoir workers following ELISA for anti-CCHFV at Al-Bawadi abattoir. The means of OD_450_ values (mean ± SEM) of positive abattoir workers with low, medium, and high antibody titer were 0.42 ± 0.032, 1.41 ± 0.10, and 2.46 ± 0.071, respectively.

The results of the comparison of the OD_450_ values of ELISA performed on abattoir workers for the detection of the CCHFV antibody are presented in Figure 3. Based on the cutoff calculated following the kit manufacturer's recommendations, 25 of the 86 abattoir workers tested positive for the CCHFV antibody, while the remaining 61 tested negatives. However, variation was observed in the OD_450_ values of the 25 seropositive abattoir workers. Hence, the OD_450_ values of the seropositive abattoir workers were classified into three groups based on the intensity of the antibody titer, namely low (OD_450_ = 0.30–0.99), medium (OD_450_ = 1.0–1.99), and high (OD_450_ ≥ 2) (Figure 3). The mean OD_450_ values (mean ± SD) of positive abattoir workers with low, medium, and high intensities were 0.42 ± 0.12, 1.41 ± 0.26, and 2.46 ± 0.16, respectively. The proportion the seropositive abattoir workers with low, medium, and high antibody titer was 52% (13/25), 28% (7/25), and 20% (5/25), respectively. Thus, the majority of the seropositive workers had low titers of CCHFV reactive antibodies.

### 3.3. Detection of CCHFV RNA by RT-qPCR in Camels and Abattoir Workers

**Figure 4:**
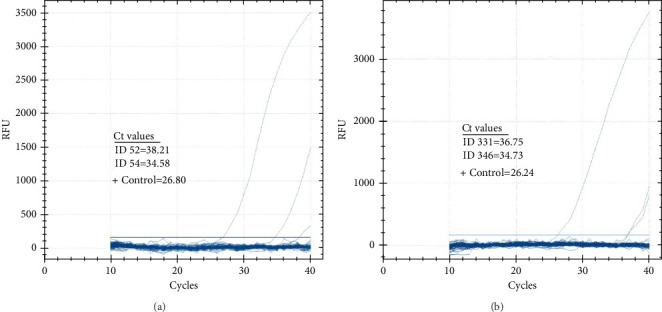
Detection of Crimean–Congo hemorrhagic fever virus (CCHFV) by reverse transcriptase quantitative real-time polymerase chain reaction (RT-qPCR) in camel sera. The sera of 393 camels were screened for CCHFV by RT-qPCR. Only four camels (Panel (A) ID: 52 and 54, and Panel (B) ID: 331 and 346) were positive. The cycle threshold (Ct) values of the four positive camels ranged from 34.58 to 38.21.

The prevalence of CCHFV RNA in camels, based on RT-qPCR, was 1% (4/393). Two of the four RT-qPCR-positive camels were also serologically positive. The cycle threshold (Ct) values of the four positive camels ranged from 34.58 to 38.21 (Figure 4A,B). On the other hand, all 86 human serum samples tested negative for CCHFV RNA by RT-qPCR.

### 3.4. Knowledge of the Abattoir Workers on Zoonotic Diseases and Transmission

**Figure 5:**
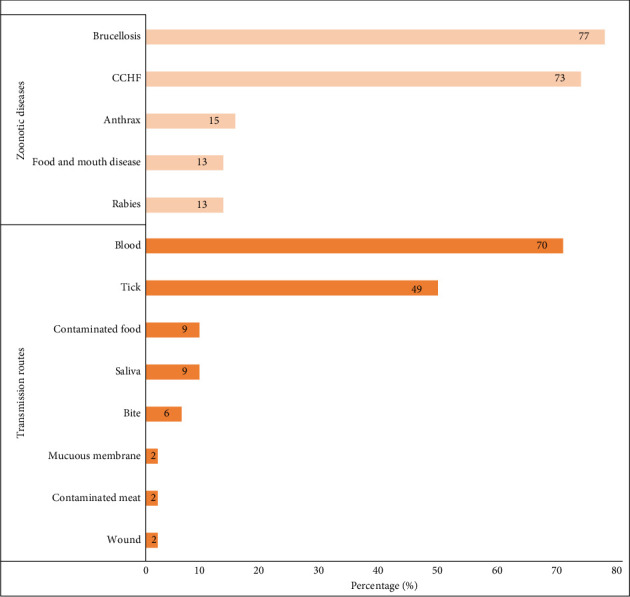
Percentage of abattoir workers reporting zoonotic diseases known to them and the routes of transmission of zoonotic diseases. Brucellosis (77%) and CCHF (73%) were known to the abattoir workers, while blood (70%) and tick bite (49%) were the routes of transmission of zoonotic diseases known by the abattoir workers.

The median overall knowledge score of the participants was 4 of 14 (IQR = 2.0, 4.0). Specifically, the median knowledge of individual zoonotic diseases was 2 of 5, whereas that of animal-to-human transmission was 2 of 9. The most commonly known zoonotic diseases were brucellosis (77%) and CCHF (73%), whereas the least commonly known diseases were foot-and-mouth disease (13%) and rabies (13%) (Figure 5). Many workers mentioned that blood (70%) and ticks (49%) were the main sources of transmission of zoonotic diseases from animals to humans (Figure 5). Butchers (4.0) had a significantly higher knowledge of zoonotic diseases than laborers (2.0) (*p*  < 0.001). In terms of knowledge differences between nationalities, Egyptians and other Africans exhibited significantly higher knowledge than other nationalities (*p*  < 0.001) (Table 4).

## 4. Discussion

The seroprevalences of CCHV infections were estimated in camels and abattoir workers in the UAE. In addition, the CCHFV RNA was investigated in the sera of both study subjects. Besides, the knowledge of the abattoir workers regarding selected zoonotic diseases and their modes of transmission.

The seroprevalence of CCHV infection in camels was 65.1%, which is high. Prior to this study, similar seroprevalence studies were published in camels from the UAE [20–23, 36]. Camp et al. [22] reported a seroprevalence of 67% from the study performed at the market located next to the Al Bawadi abattoir where the present study was conducted. The market serves as the major source of the camels for slaughter at the Al Bawadi abattoir, which could suggest that there is a similar ratio of infected camels over time, showing an ongoing focus on transmission of CCHFV. Thus, the results of the present and previous studies could suggest that the potential role of camels as reservoirs for CCHFV and their significance in the epidemiology and zoonotic transmission of the disease in the country. In this study, no difference in seroprevalence was observed between camels of different sexes, age groups, or body conditions of the study camels. Similarly, high seroprevalence values were reported in camels from Mauritania [37] and Tunisia [38]. On the other hand, moderate seroprevalence values were reported in camels from Iraq, Egypt, Oman, and Sudan [39].

Although the seroprevalence recorded in this study was high, only viral RNA was detected only in 1% of the study camels. The presence of RNA could suggest ongoing infection, whereas CCHFV reactive antibodies indicate exposure to CCHFV [40]. The low prevalence of actively circulating virus in seropositive camels could be due to the short duration of viremia [41, 42], and thus, the seropositivity could be associated with past exposure. In addition, as observed in the result of this study, the majority of the seropositive camels exhibited strong CCHFV reactive antibodies titer, which could clear the CCHFV infection from the camels. Viral RNA was detected in two seronegative camels. This could be due to early infection where the camels did not reach the dates of antibodies production against the infection, or it could also be due to immunosuppression in these two camels [41]. The observation of viral RNA or antibodies could suggest that CCHFV may emerge as a zoonotic pathogen threatening the public health [40]. Therefore, active surveillance is necessary to detect hot spots of CCHFV infection in camels and other domestic animals so that the results of the surveys can inform public health authorities for proactive responses.

Perveen and Khan [43] published a systematic review of CCHFV in Arab countries and reported a pooled seroprevalence of 29.0% in camels. Thus, the seroprevalence values reported by different researchers vary, ranging from the lowest (7.4%) to the highest (89.7%). Such variations in CCHFV seroprevalence in camels could be due to (1) differences in serological tests used by the studies, (2) variation in the abundance of the tick vector, (3) season of the year, (4) presence or absence of tick control methods, (5) suitability of the ecology of the study area for tick reproduction, (6) presence of wildlife that could serve as symptomatic reservoirs of the virus, and (7) nutritional and immunological status of the camels.

The seroprevalence of CCHFV infection was 29.1% in abattoir workers, although CCHFV RNA was not detected from any of the abattoir workers, which could exclude the presence of active infection in the abattoir workers. In addition, previous studies indicated that CCHFV infection is characterized by a low and short duration of viremia, and the chance of detecting viral RNA is low [34, 41]. Furthermore, the virus is cleared by anti-CCHFV antibodies approximately a week after the initial infection, leading to the absence of viral RNA in circulation [44–46]. However, the high seroprevalence could be associated with past exposure or infection of abattoir workers to infected camels and other animals in the slaughterhouse.

A few studies were published on human CCHFV infection in the UAE with different conclusions with regard to the endemicity of the infection in the country. According to Al Dabal et al. [21], CCHF is not endemic to the UAE, despite the two small-scale outbreaks (1979 and 1994–1995) and the two cases reported from the UAE reported in 2010 [20, 21, 36]. The first outbreak in the UAE occurred in 1979 in Dubai, when an index case transmitted the infection to five healthcare workers [36]. During that outbreak, the index case and two of the five healthcare workers died [36]. The second outbreak of CCHF in the UAE occurred from 1994 to 1995 in which 35 suspected cases were identified among livestock market employees, abattoir workers, and animal skin processors [20]. The fatality rate was 62% during this outbreak [20]. In addition to these outbreaks, two cases were reported in Dubai in 2010 [21]. Although Al Dabal et al. [21] indicated that CCHF was not endemic to the UAE, Camp et al. [22] suggested the opposite. The latter authors indicated the endemicity of CCHF in the UAE based on the detection of CCHFV reactive antibodies in the sera of camels and the detection of viral RNA in the sera of seropositive camels and ticks attached to these camels. In addition to the above reports from the UAE, a recent study detected CCHFV RNA in 16 of 238 dromedary camels using nasal swab screening and RT-PCR [23].

Similarly, high seroprevalence was reported in human occupational contacts in Oman [25]. Human CCHF cases were observed in Oman between 2011 and 2017 and the main risk groups were butchers [47]. The total number of human CCHF cases recorded in Oman was 88, of which 32 patients died [47]. In addition, human cases and deaths due to CCHF from other countries were reviewed and published earlier [43]. Accordingly, human cases and deaths were reported from Iraq [39, 48–50], Saudi Arabia [24], Mauritania [51–54], Sudan [54–57], Tunisia and Kuwait [58, 59].

In this study, a difference in the magnitude of CCHFV reactive antibodies was observed between the seropositive camels and seropositive abattoir workers. A large proportion (45%) of the seropositive camels had high CCHFV reactive antibodies titer, while a large proportion (52%) of the seropositive abattoir workers had low CCHFV reactive antibodies titer. Although this study is a cross-sectional study and may not lead to draw a conclusive finding, the observation of such difference could be one of the reasons for the difference in the severity of CCHFV infection in camels and humans (abattoir workers). Authors indicated [60] that in addition to innate immunity, successful control of CCHFV infection requires an effective adaptive immune response, including strong CCHFV reactive antibodies. It was documented that low or absent antibody responses are frequently associated with poor disease outcomes in CCHFV-infected humans and mice [44, 60–62]. Furthermore, it was observed that severe CCHF cases had minimal humoral immune response while, conversely, survivors developed CCHFV-specific humoral and cellular immunity [8]. Furthermore, Cohen et al. [63] reported the significance of strong antibody response against various clades of CCHFV in a Ugandan human cohort study. All these studies indicate the significance of CCHF reactive antibodies for controlling of CCHFV infection. Thus, the high antibodies titer in camels could be the reason the subclinical infection in camels.

Comparison of the seroprevalence of CCHFV infection among the abattoirs workers of different nationalities did not reveal the absence of difference among nationalities. This could be due to the fact that all abattoir workers had equal chances of exposure to infected animals and also used similar types of PPE. All the abattoir workers reported using PPE. In contrast, a relatively higher seroprevalence of CCHFV was observed in the abattoir workers with a longer duration of working in the abattoir. This could be due to the increased chance of exposure to infected animals or ticks.

Furthermore, the knowledge of abattoir workers regarding zoonotic diseases and their routes of transmission to humans varied among individuals of different nationalities. Five diseases, namely brucellosis, CCHF, anthrax, foot-and-mouth disease, and rabies, were reported as zoonotic diseases known by the abattoir workers. Brucellosis and CCHF were the zoonotic diseases most widely recognized by the abattoir workers. This could be associated with the creation of awareness among abattoir workers about brucellosis and CCHF because of the occurrence of these diseases in the UAE. Furthermore, abattoir workers reported knowing that blood and ticks are common routes of transmission of zoonotic diseases to humans.

CCHFV is geographically widespread because of its tick vector, which transports the virus over long distances and can even be transcontinental. Trading livestock infested with infected ticks is an important route for the spread of the virus. The endemic transmission cycle of the virus involves livestock and tick species of the genus *Hyalomma* [64]. The epidemic cycle occurs when infected ticks accidentally bite humans or when humans are exposed to the blood or tissues of infected animals [22]. Previous studies have detected CCHFV in different species of *Hyalomma* tick in the UAE [20]. Khan et al. [20] reported the detection of anti-CCHFV antibodies in *Hyalomma* (*H*) *impelatum*, *H. excavatum*, and *H. anatolicum* during the 1994–1995 outbreak in the UAE. Furthermore, Rodriguez et al. [65] detected CCHFV nucleic acids in *Hyalomma* spp. from the UAE. Similarly, Camp et al. [22] and Khalafalla et al. [23] detected anti-CCHFV antibodies and CCHFV nucleic acids in *H. dromedarii* in the UAE, respectively.

Finally, this study has some limitations. The limitations of this study include the inability to investigate viral RNA in the tick vectors. Moreover, the study could not identify the origin of the camels the merchants could not provide this information either to the research team or to the abattoir workers.

## 5. Conclusion

In conclusion, the seroprevalence of CCHFV infection was high in the slaughtered camels and abattoir workers at the Al-Bawadi Abattoir. However, viral RNA was detected in only 1% of the study camels, while it was not detected from the abattoir workers. It is known that CCHFV infection rarely causes clinical disease in animals, including camels, while on the other hand causes a significant public health impact with high morbidity and mortality rates. Nonetheless, there is no approved vaccine for the control of CCHF in humans and treatment is restricted to the treatment of the symptoms [3]. In addition, it is difficult to control CCHF infection in animals and ticks as the tick-animal-tick cycle usually goes unnoticed, and the infection in domestic animals is usually not apparent.

Health education and awareness creation on prevention and behavioral measures are most important in order to enhance public risk perception in order to decrease the probability of infection. To this effect, the identification of endemic foci is crucial for the implementation of public health measures. Once endemic areas are identified using serological screening of domestic ruminants and camels, control and preventive measures should be implemented. According to WHO [3] recommendation, the control and preventive measures include (1) reducing the risk of tick-to-human transmission, (2) reducing the risk of animal-to-human transmission, and (3) reducing the risk of animal-to-human transmission. Therefore, since CCHF infection is endemic in the UAE, a comprehensive seroprevalence surveillance of CCHFV in different animal species and human occupational risk groups is recommended in order to identify high-burden foci and then thereafter implement the above-mentioned control and preventive measures.

## Figures and Tables

**Table 1:** Association of the seroprevalence of CCHFV infection with the demographic characteristics of the camels based on binary logistic regression analyses.

Factor	Categories	Positive	Total	Univariable odds ratio (OR)	95% CI	Multivariable OR	95% CI
Sex	Male	68 (69.4%)	98	0.78	0.48, 1.27	0.76	0.45, 1.29
	Female	188 (63.7%)	295	1	—	1	—

Age	<3	76 (65%)	117	1.14	0.66, 1.97	0.85	0.44, 1.63
	3–5	76 (68%)	112	1.01	0.57, 1.79	0.76	0.40, 1.42
	5–7	62 (65.3%)	95	0.84	0.45, 1.55	0.82	0.43, 1.58
	7–12	42 (61%)	69	1	—	1	—

Body condition	Thin	27 (77.1%)	35	0.50	0.22, 1.14	0.61	0.24, 1.55
	Medium	171 (62.6%)	273	0.64	0.26, 1.59	1.28	0.76, 2.15
	Fat	58 (68.2%)	85	1	—	1	—
	Total	256 (65.1)	393	—	—	—	—

**Table 2:** Demographic characteristics of the abattoir workers (*N* = 86).

Characteristic	*N* (%)
Age in years, median (IQR)	36 (30, 41)
Occupation	—
Butcher	71 (83%)
Laborer	13 (15%)
Supervisor	2 (2.3%)
Years of experience, median (IQR)	8.0 (6.0, 14.0)
Nationality	—
Egypt	11 (12.9%)
Bangladesh	20 (23.5%)
Pakistan	36 (42.4)
Other Asians^a^	11 (12.9%)
Other Africans^b^	7 (8.2%)
Affected by zoonotic diseases	—
No	82 (95%)
Yes	4 (4.7%)
Knew a co-worker affected with zoonotic disease	—
No	79 (92%)
Yes	7 (8.1%)

^a^Includes Nepal (*n* = 5), Sri Lanka (*n* = 4), and Afghanistan (*n* = 2).

^b^Includes Sudan (*n* = 4), Morocco (*n* = 2), and Ethiopia (*n* = 1).

**Table 3:** Association of the seroprevalence of Crimean–Congo hemorrhagic fever (CCHF) with abattoir workers' characteristics.

Characteristic	CCHF serology test result		*p*-Value^a^
	Positive (*N* = 25)	Negative (*N* = 60)	
Overall prevalence (95% CI)	29.1 (20.3–40.4)	—	—
Age in years, median (IQR)	35 (30, 47)	36 (30, 40)	0.589
Job position	—	—	>0.999
Butcher	20 (29%)	50 (71%)	—
Laborer	4 (31%)	9 (69%)	—
Years of experience, median (IQR)	10.0 (6.0, 14.0)	7.5 (5.0, 14.0)	0.299
Nationality	—	—	0.294
Egypt	6 (54.5%)	5 (45.5%)	—
Bangladesh	5 (25%)	15 (75%)	—
Pakistan	8 (22.2%)	28 (77.8%)	—
Other Asians^b^	4 (40%)	6 (60%)	—
Other Africans^c^	2 (28.6%)	5 (71.4%)	—
Affected by zoonotic diseases	—	—	>0.999
No	24 (30%)	57 (70%)	—
Yes	1 (25%)	3 (75%)	—
Knew a co-worker affected with zoonotic disease	—	—	>0.999
No	23 (29%)	55 (71%)	—
Yes	2 (29%)	5 (71%)	—

^a^Pearson's chi-square test, Fisher's exact test, and Mann–Whitney *U* test were used.

^b^Includes Nepal (*n* = 5), Sri Lanka (*n* = 4), and Afghanistan (*n* = 2).

^c^Includes Sudan (*n* = 4), Morocco (*n* = 2), and Ethiopia (*n* = 1).

**Table 4:** Factors associated with participants' knowledge of zoonotic diseases and their transmission.

Characteristic	Knowledge score, median (IQR)	*p*-Value^a^
Overall knowledge score	—	—
Median (IQR)	4.0 (2.0, 4.0)	—
Minimum, maximum	2.0, 8.0	—
Maximum possible score	14.0	—
Occupation	—	<0.001
Butcher	4.0 (2.0, 4.0)	—
Laborer	2.0 (2.0, 2.0)	—
Nationality	—	<0.001
Egypt	4.0 (4.0, 6.0)	—
Bangladesh	3.0 (2.0, 4.0)	—
Pakistan	2.5 (2.0, 4.0)	—
Other Asians^b^	2.0 (2.0, 3.0)	—
Other Africans^c^	5.0 (4.0, 5.0)	—
Affected by zoonotic diseases	—	0.302
No	3.50 (2.0, 4.0)	—
Yes	4.00 (4.0, 4.0)	—
Knew a co-worker affected with zoonotic disease	—	0.874
No	4.0 (2.0, 4.0)	—
Yes	4.0 (3.0, 4.0)	—

^a^Mann–Whitney *U* test and Kruskal–Wallis ANOVA test were used.

^b^Includes Nepal (*n* = 5), Sri Lanka (*n* = 4), and Afghanistan (*n* = 2).

^c^Includes Sudan (*n* = 4), Morocco (*n* = 2), and Ethiopia (*n* = 1).

## Data Availability

The data that support the findings of this study are available from the corresponding author upon reasonable request.

## References

[1] Hawman D. W., Feldmann H. (2023). Crimean-Congo Haemorrhagic Fever Virus. *Nature Reviews Microbiology*.

[2] WOAH - World Organisation for Animal Health (2024). Crimean-Congo Haemorrhagic Fever [Online]. https://www.woah.org/en/disease/crimean-congo-haemorrhagic-fever/.

[3] WHO (2025). Crimean-Congo Haemorrhagic Fever, World Health Organization (WHO) Report [Online]. https://www.who.int/health-topics/crimean-congo-haemorrhagic-fever.

[4] Frank M. G., Weaver G., Raabe V. (2024). Crimean-Congo Hemorrhagic Fever Virus for Clinicians—Diagnosis, Clinical Management, and Therapeutics. *Emerging Infectious Diseases*.

[5] Grard G., Drexler J. F., Fair J. (2011). Re-Emergence of Crimean-Congo Hemorrhagic Fever Virus in Central Africa. *PLoS Neglected Tropical Diseases*.

[6] Papa A., Tzala E., Maltezou H. C. (2011). Crimean-Congo Hemorrhagic Fever Virus, Northeastern Greece. *Emerging Infectious Diseases*.

[7] Swanepoel R., Paweska J. T., Palmer S. R., Soulsby Lord, Torgerson Paul, Brown David W. G. (2011). Crimean-Congo Haemorrhagic Fever. *Oxford Textbook of Zoonoses: Biology, Clinical Practice, and Public Health Control*.

[8] Bente D. A., Forrester N. L., Watts D. M., McAuley A. J., Whitehouse C. A., Bray M. (2013). Crimean-Congo Hemorrhagic Fever: History, Epidemiology, Pathogenesis, Clinical Syndrome and Genetic Diversity. *Antiviral Research*.

[9] Gargili A., Estrada-Peña A., Spengler J. R., Lukashev A., Nuttall P. A., Bente D. A. (2017). The Role of Ticks in the Maintenance and Transmission of Crimean-Congo Hemorrhagic Fever Virus: A Review of Published Field and Laboratory Studies. *Antiviral Research*.

[10] Whitehouse C. A. (2004). Crimean-Congo Hemorrhagic Fever. *Antiviral Research*.

[11] Hoogstraal H. (1979). The Epidemiology of Tick-Borne Crimean-Congo Hemorrhagic Fever in Asia, Europe, and Africa. *Journal of Medical Entomology*.

[12] Messina J. P., Pigott D. M., Golding N. (2015). The Global Distribution of Crimean-Congo Hemorrhagic Fever. *Transactions of The Royal Society of Tropical Medicine and Hygiene*.

[13] Lindeborg M., Barboutis C., Ehrenborg C. (2012). Migratory Birds, Ticks, and Crimean-Congo Hemorrhagic Fever Virus. *Emerging Infectious Diseases*.

[14] Rainey T., Occi J. L., Robbins R. G., Egizi A. (2018). Discovery of *Haemaphysalis longicornis* (Ixodida: Ixodidae) Parasitizing a Sheep in New Jersey, United States. *Journal of Medical Entomology*.

[15] Grandi G., Chitimia-Dobler L., Choklikitumnuey P. (2020). First Records of Adult Hyalomma Marginatum and *H. rufipes* Ticks (Acari: Ixodidae) in Sweden. *Ticks and Tick-borne Diseases*.

[16] Garrison A. R., Alkhovsky S. V., Avšič-Županc T. (2020). ICTV Virus Taxonomy Profile: Nairoviridae. *Journal of General Virology*.

[17] Wang Y., Dutta S., Karlberg H. (2012). Structure of Crimean-Congo Hemorrhagic Fever Virus Nucleoprotein: Superhelical Homo-Oligomers and the Role of Caspase-3 Cleavage. *Journal of Virology*.

[18] Mishra A. K., Hellert J., Freitas N. (2022). Structural Basis of Synergistic Neutralization of Crimean-Congo Hemorrhagic Fever Virus by Human Antibodies. *Science*.

[19] Baskerville A., Satti A., Murphy F. A., Simpson D. I. (1981). Congo-Crimean Haemorrhagic Fever in Dubai: Histopathological Studies. *Journal of Clinical Pathology*.

[20] Khan A. S., Maupin G. O., Rollin P. E. (1997). An Outbreak of Crimean-Congo Hemorrhagic Fever in the United Arab Emirates, 1994–1995. *The American Journal of Tropical Medicine and Hygiene*.

[21] Mohamed AL Dabal L., Rahimi Shahmirzadi M. R., Baderldin S. (2016). Crimean-Congo Hemorrhagic Fever in Dubai, United Arab Emirates, 2010: Case Report. *Iranian Red Crescent Medical Journal*.

[22] Camp J. V., Kannan D. O., Osman B. M. (2020). Crimean-Congo Hemorrhagic Fever Virus Endemicity in United Arab Emirates, 2019. *Emerging Infectious Diseases*.

[23] Khalafalla A. I., Li Y., Uehara A. (2021). Identification of a Novel Lineage of Crimean-Congo Haemorrhagic Fever Virus in Dromedary Camels, United Arab Emirates. *Journal of General Virology*.

[24] El-Azazy O. M. E., Scrimgeour E. M. (1997). Crimean-Congo Haemorrhagic Fever Virus Infection in the Western Province of Saudi Arabia. *Transactions of the Royal Society of Tropical Medicine and Hygiene*.

[25] Williams R. J., Al-Busaidy S., Mehta F. R. (2000). Crimean-Congo Haemorrhagic Fever: A Seroepidemiological and Tick Survey in the Sultanate of Oman. *Tropical Medicine & International Health*.

[26] Akuffo R., Brandful J. A. M., Zayed A. (2016). Crimean-Congo Hemorrhagic Fever Virus in Livestock Ticks and Animal Handler Seroprevalence at an Abattoir in Ghana. *BMC Infectious Diseases*.

[27] Adham D., Abazari M., Moradi-Asl E., Abbasi-Ghahramanloo A. (2021). Pattern of Crimean-Congo Hemorrhagic Fever Related High Risk Behaviors Among Iranian Butchers and Its Relation to Perceived Self-Efficacy. *BMC Public Health*.

[28] Adamu A. M., Onoja A. B., Ugbodu V. E. (2024). Investigating Crimean-Congo Haemorrhagic Fever Virus Seropositivity in Camels and Human Behavioural Risks in an Abattoir in Nigeria. *Epidemiology and Infection*.

[29] Sheek-Hussein M., Zewude A., Abdullahi A. S. (2025). One Health Approach Based Descriptive Study on *Coxiella burnetii* Infections in Camels and Abattoir Workers in the United Arab Emirates. *Scientific Reports*.

[30] Faye B., Bengoumi M., Cleradin A., Tabarani A., Chilliard Y. (2001). Body Condition Score in Dromedary Camel: A Tool for Management of Reproduction. *Emirates Journal of Food and Agriculture*.

[31] Menchetti L., Faye B., Padalino B. (2021). New Animal-Based Measures to Assess Welfare in Dromedary Camels. *Tropical Animal Health and Production*.

[32] Wagener M. G., Schregel J., Ossowski N., Trojakowska A., Ganter M., Kiene F. (2023). The Influence of Different Examiners on the Body Condition Score (BCS) in South American Camelids-Experiences From a Mixed Llama and Alpaca Herd. *Frontiers in Veterinary Science*.

[33] Bello A., Sonfada M. L., Umar A. A. (2013). Age Estimation of Camel in Nigeria Using Rostral Dentition. *Scientific Journal of Animal Science*.

[34] Raabe V. N., Kraft C. S. (2020). Diagnostic Testing for Crimean-Congo Hemorrhagic Fever. *Journal of Clinical Microbiology*.

[35] Wölfel R., Paweska J. T., Petersen N. (2007). Virus Detection and Monitoring of Viral Load in Crimean-Congo Hemorrhagic Fever Virus Patients. *Emerging Infectious Diseases*.

[36] Suleiman M. N., Muscat-Baron J. M., Harries J. R. (1980). Congo/Crimean Haemorrhagic Fever in Dubai. An Outbreak at the Rashid Hospital. *Lancet*.

[37] Schulz A., Barry Y., Stoek F. (2021). Crimean-Congo Hemorrhagic Fever Virus Antibody Prevalence in Mauritanian Livestock (cattle, Goats, Sheep and Camels) Is Stratified by the Animal’s Age. *PLOS Neglected Tropical Diseases*.

[38] Bouaicha F., Eisenbarth A., Elati K. (2021). Epidemiological Investigation of Crimean-Congo Haemorrhagic Fever Virus Infection Among the One-Humped Camels (*Camelus dromedarius*) in Southern Tunisia. *Ticks and Tick-borne Diseases*.

[39] Tantawi H. H., Shony M. O., Al-Tikriti S. K. (1981). Antibodies to Crimean-Congo Haemorrhagic Fever Virus in Domestic Animals in Iraq: A Seroepidemiological Survey. *International Journal of Zoonoses*.

[40] Nasirian H. (2019). Crimean-Congo Hemorrhagic Fever (CCHF) Seroprevalence: A Systematic Review and Meta-Analysis. *Acta Tropica*.

[41] Spengler J. R., Estrada-Peña A., Garrison A. R. (2016). A Chronological Review of Experimental Infection Studies of the Role of Wild Animals and Livestock in the Maintenance and Transmission of Crimean-Congo Hemorrhagic Fever Virus. *Antiviral Research*.

[42] Balinandi S., von Brömssen C., Tumusiime A. (2021). Serological and Molecular Study of Crimean-Congo Hemorrhagic Fever Virus in Cattle From Selected Districts in Uganda. *Journal of Virological Methods*.

[43] Perveen N., Khan G. (2022). Crimean-Congo Hemorrhagic Fever in the Arab World: A Systematic Review. *Frontiers in Veterinary Science*.

[44] Shepherd A. J., Swanepoel R., Leman P. A. (1989). Antibody Response in Crimean-Congo Hemorrhagic Fever. *Clinical Infectious Diseases*.

[45] Kaya S., Elaldi N., Kubar A. (2014). Sequential Determination of Serum Viral Titers, Virus-Specific IgG Antibodies, and TNF-*α*, IL-6, IL-10, and IFN-*γ* Levels in Patients With Crimean-Congo Hemorrhagic Fever. *BMC Infectious Diseases*.

[46] Ergunay K., Kocak Tufan Z., Bulut C., Kinikli S., Demiroz A. P., Ozkul A. (2014). Antibody Responses and Viral Load in Patients With Crimean-Congo Hemorrhagic Fever: A Comprehensive Analysis During the Early Stages of the Infection. *Diagnostic Microbiology and Infectious Disease*.

[47] Al-Abri S. S., Hewson R., Al-Kindi H. (2019). Clinical and Molecular Epidemiology of Crimean-Congo Hemorrhagic Fever in Oman. *PLOS Neglected Tropical Diseases*.

[48] Al-Tikriti S. K., Al-Ani F., Jurji F. J. (1981). Congo/Crimean Haemorrhagic Fever in Iraq. *Bulletin of the World Health Organization*.

[49] Majeed B., Dicker R., Nawar A., Badri S., Noah A., Muslem H. (2012). Morbidity and Mortality of Crimean-Congo Hemorrhagic Fever in Iraq: Cases Reported to the National Surveillance System, 1990–2010. *Transactions of the Royal Society of Tropical Medicine and Hygiene*.

[50] Ibrahim A., Ibrahim K., Omer M. (2014). Crimean Congo Hemorrhagic Fever Management in Erbil During 2010–2011. *European Scientific Journal*.

[51] Gonzalez J.-P., LeGuenno B., Guillaud M., Wilson M. L. (1990). A Fatal Case of Crimean-Congo Haemorrhagic Fever in Mauritania: Virological and Serological Evidence Suggesting Epidemic Transmission. *Transactions of the Royal Society of Tropical Medicine and Hygiene*.

[52] Nabeth P., Thior M., Faye O., Simon F. (2004). Human Crimean-Congo Hemorrhagic Fever, Sénégal. *Emerging Infectious Diseases*.

[53] Boushab B. M., Kelly M., Kébé H., Bollahi M. A., Basco L. K. (2020). Crimean-Congo Hemorrhagic Fever, Mauritania. *Emerging Infectious Diseases*.

[54] Temur A. I., Kuhn J. H., Pecor D. B., Apanaskevich D. A., Keshtkar-Jahromi M. (2021). Epidemiology of Crimean-Congo Hemorrhagic Fever (CCHF) in Africa—Underestimated for Decades. *The American Journal of Tropical Medicine and Hygiene*.

[55] Aradaib I. E., Erickson B. R., Mustafa M. E. (2010). Nosocomial Outbreak of Crimean-Congo Hemorrhagic Fever, Sudan. *Emerging Infectious Diseases*.

[56] Elata A. T., Karsany M. S., Elageb R. M. (2011). A Nosocomial Transmission of Crimean-Congo Hemorrhagic Fever to an Attending Physician in North Kordufan, Sudan. *Virology Journal*.

[57] Abdelhakam H. A. A., Taha M. A. (2014). Crimean-Congo Hemorrhagic Fever (CCHF) in Southern Kordofan. *Sudanese Journal of Paediatrics*.

[58] Al-Nakib W., Lloyd G., El-Mekki A., Platt G., Beeson A., Southee T. (1984). Preliminary Report on Arbovirus-Antibody Prevalence Among Patients in Kuwait: Evidence of Congo/Crimean Virus Infection. *Transactions of the Royal Society of Tropical Medicine and Hygiene*.

[59] Wasfi F., Dowall S., Ghabbari T. (2016). Sero-Epidemiological Survey of Crimean-Congo Hemorrhagic Fever Virus in Tunisia. *Parasite*.

[60] Rodriguez S. E., Hawman D. W., Sorvillo T. E. (2022). Immunobiology of Crimean-Congo Hemorrhagic Fever. *Antiviral Research*.

[61] Ergonul O., Celikbas A., Baykam N., Eren S., Dokuzoguz B. (2006). Analysis of Risk-Factors Among Patients With Crimean-Congo Haemorrhagic Fever Virus Infection: Severity Criteria Revisited. *Clinical Microbiology and Infection*.

[62] Saksida A., Duh D., Wraber B., Dedushaj I., Ahmeti S., Avšič-Županc T. (2010). Interacting Roles of Immune Mechanisms and Viral Load in the Pathogenesis of Crimean-Congo Hemorrhagic Fever. *Clinical and Vaccine Immunology*.

[63] Cohen C. A., Balinandi S., Kuehne A. I. (2025). A Longitudinal Analysis of Memory Immune Responses in Convalescent Crimean-Congo Hemorrhagic Fever Survivors in Uganda. *Journal of Infectious Diseases*.

[64] Spengler J. R., Bergeron É., Spiropoulou C. F. (2019). Crimean-Congo Hemorrhagic Fever and Expansion From Endemic Regions. *Current Opinion in Virology*.

[65] Rodriguez L. L., Maupin G. O., Ksiazek T. G. (1997). Molecular investigation of a multisource outbreak of Crimean-Congo hemorrhagic fever in the United Arab Emirates. *American Journal of Tropical Medicine and Hygeine*.

